# Small Esophageal Varices in Patients with Cirrhosis—Should We Treat Them?

**DOI:** 10.1007/s11901-018-0420-z

**Published:** 2018-11-07

**Authors:** Thomas Reiberger, Theresa Bucsics, Rafael Paternostro, Nikolaus Pfisterer, Florian Riedl, Mattias Mandorfer

**Affiliations:** 1 Vienna Hepatic Hemodynamic Lab, Medical University of Vienna, Vienna, Austria; 2 Division of Gastroenterology & Hepatology, Department of Medicine III, Medical University of Vienna, Vienna, Austria; 3 Division of Gastroenterology, Krankenanstalt Rudolfstiftung, Vienna, Austria; 4 Division of Gastroenterology, Medicine II, Universitätsklinikum St. Pölten, St. Pölten, Austria

**Keywords:** Small varices, Portal hypertension, Cirrhosis, Variceal bleeding, Low-risk varices

## Abstract

**Purpose of Review:**

The natural history and classification systems of small varices (≤ 5 mm in diameter) in cirrhotic patients with portal hypertension are summarized. Studies that assessed the course of and therapeutic intervention for small varices are discussed.

**Recent Findings:**

Current non-invasive methods show suboptimal sensitivity to detect small varices in patients with cirrhosis. Next to etiological therapy, hepatic venous pressure gradient (HVPG)-guided non-selective betablocker or carvedilol treatment has shown to impact on natural history of small varices.

**Summary:**

The main therapeutic focus in cirrhotic patients with small varices is the cure of the underlying etiology. The optimal management of small varices should include measurement of HVPG. A pharmacological decrease in HVPG by non-selective betablocker therapy of ≥ 10% reduces the risk of progression to large varices, first variceal bleeding, and hepatic decompensation. If HVPG is not available, we would recommend carvedilol 12.5 mg q.d. for treatment of small varices in compensated patients without severe ascites. Only if small esophageal varices (EV) are not treated or in hemodynamic non-responders, follow-up endoscopies should be performed in 1–2 years of intervals considering the activity of liver disease or if hepatic decompensation occurs.

## Introduction

Patients with cirrhosis (i.e., advanced chronic liver disease, ACLD) might develop clinically significant portal hypertension (CSPH) with hepatic venous pressure gradient (HVPG) ≥ 10 mmHg. With increasing severity of portal hypertension, patients are more likely to suffer from first variceal bleeding and recurrent variceal bleeding from esophageal varices (EV). The beneficial role of non-selective betablockers (NSBB) in combination with endoscopic band ligation (EBL) in preventing recurrent variceal bleeding (secondary prophylaxis) has been clearly demonstrated in several trials [1] and meta-analyses [2]. In the setting of primary prophylaxis, there is strong evidence for a beneficial role of NSBB if EV are medium-large sized [3•, 4•], but the impact of NSBB is less clear in patients with small EV. While there is no conclusive evidence that NSBB treatment reduces the risk of variceal bleeding or mortality in patients with small low-risk varices who have not bled [5], the absence of evidence should not be mistaken for evidence of absence. Most trials in patients with no/small varices were not sufficiently powered to detect favorable effects of NSBB on hard endpoints such as variceal bleeding or hepatic decompensation, which occur less commonly in this group of patients [6]. This is the reason why well-designed studies on clinically meaningful surrogate endpoints (e.g., variceal growth) are particularly relevant in this setting. Previously, there were two important trials with somewhat contradictory results: While a randomized controlled trial (RCT) by Merkel et al. [7] demonstrated that conventional NSBB (nadolol) therapy is effective in preventing the progression from small to large varices in patients who have not bled, another RCT by Sarin and co-workers [8] using propranolol reported no effect. In this review on small EV, we aim to summarize important non-invasive and invasive diagnostic aspects, pathophysiological considerations, and available evidence for pharmacological therapy to guide treatment decisions in daily clinical practice.

### Classification of EV: Size, Location, Presence of Red Spots, Low Versus High Risk

In CSPH, sub-epithelial longitudinal veins might enlarge to form varices in the esophagus. With increasing size and with rising intravascular pressure, they are more likely to rupture. Screening endoscopy is traditionally recommended to be performed in all patients after the diagnosis of cirrhosis. To timely initiate prophylaxis for variceal bleeding, early detection and specific classification of EV are major goals in the management of patients with ACLD [9]. Reports of gastroduodenoscopy performed in patients with cirrhosis must include information about the absence/presence of varices, their size, their location, and presence of red spot signs. The latter indicates sites where the variceal wall is thinning, and thus represents a sign for increased risk of EV rupture and bleeding.

Historically, the first endoscopic classification was developed by Brick and Palmer in 1964 [10]. Over time, other classifications were presented, with several ways to describe the size/grade of varices [11–13]. Based on these classifications, varices were described as grades I to IV, or more intuitively, as small, medium, or large. The presence of risk factors for variceal rupture was described as red wale signs, cherry spots, red spots, or by similar terminology. The recently updated Austrian Billroth III consensus simplified the grading of EV size just into small (< 5 mm) and large (≥ 5 mm) in diameter [14].

For risk stratification, additionally to EV size, identification of red spot signs and calculation of Child-Pugh score are crucial, since these endoscopic findings and the Child-Pugh score influence the risk of bleeding and bleeding-related mortality [14, 15]. Importantly, EV can be classified as low-risk (< 5 mm without endoscopic risk factors in patients with Child-Pugh A/B) or high-risk (all EV of ≥ 5 mm in diameter or small EV showing red spot signs or small EV in patients with Child-Pugh C) varices regarding the respective risk for variceal bleeding [14, 16]. Additionally, number of varices and localization should be thoroughly described. Due to the lack of sufficient endoscopic measurement systems, size assessment and endoscopic treatment of EV demand high level of experience and should therefore be performed in specialized centers.

### Non-invasive Screening for Any Esophageal Varices and “Varices Needing Treatment” (Table 1)

Liver stiffness measured by transient elastography (TE) is a well-validated tool for the assessment of liver fibrosis [39]. Recently, the term “compensated advanced chronic liver disease” (cACLD) has been introduced by the Baveno VI consortium [3•] with liver stiffness measurement (LSM) values > 15 kPa being highly suggestive of cACLD. In clinical practice, a qualitative assessment of EV (size as well as presence or absence of red spot signs) is helpful to determine the optimal treatment strategy. Therefore, the term “varices needing treatment (VNT)” has been defined as follows: Varices of medium/large size (> 5-mm diameter), or small varices with red spot signs [3•]. According to the Baveno VI criteria, patients with TE values < 20 kPa and a platelet count ≥ 150 G/L have a very low probability for the presence of VNT. Thus, the combined TE < 20 kPa and platelet count > 150 G/L can be used to avoid futile screening endoscopies in patients with cACLD [3•].

**Table 1:** Summary of prospective studies and meta-analyses on the diagnostic value of transient elastography for the diagnosis of any varices and “varices needing treatment”

Author, journal, year	Design and etiology	*N* patients overall *N* patients with any EV (%) *N* patients with VNT (%)	TE-cutoffs and AUC any EV/VNT	Main conclusions (specificity/sensitivity, PPV/NPV)	Additional parameters used in the study and comments
Foucher, *Gut*, 2006 [17]	Prospective Mixed etiology	*n* = 144 patients with F3/F4 fibrosis *n* with any EV: n/a *n* = 42 (29%)/85 (59%) with VNT	LSM: Any: n/a VNT 27.5 kPa, AUC 0.73	LSM: Any: n/a VNT: Sens. 88%, Spec. 53%, PPV 45%, NPV 90%	• The main focus was diagnosis of fibrosis stages using TE
Kazemi, *J Hepatol*, 2006 [18]	Prospective Mixed etiology	*N* = 165 patients analyzed *n* = 74 (44.8%) with EV *n* = 47 (28.5%) with VNT	LSM: Any 13.9 kPa, AUC 0.84 VNT 19.0 kPa, AUC 0.83,	LSM: Any: Sens. 92%, Spec. 39%, PPV 55%, NPV 85% VNT Sens. 89%, Spec. 59%, PPV 47%, NPV 93%	*•* Parameters: PLT, spleen diameter, PLT, LSM/spleen size
Vizzutti, *Hepatology*, 2007 [19]	Prospective Etiology: HCV	*n* = 61 patients analyzed *n* = 30 (49.2%) with esophageal EV *n* = 18 (38.2%) with large EV/VNT	LSM: Any 17.6 kPa, AUC 0.76 VNT 27.4 kPa, AUC 0.76	LSM: Any Sens. 90%, spec. 43%, PPV 77%, NPV 66% VNT Sens. 70%, spec. 78%, PPV 90%, NPV 55%	
Bureau, *Aliment Pharmacol Ther*, 2008 [20]	Prospective Mixed etiology	*n* = 150 patients analyzed *n* = 64 (42.6%) with EV *n* = 43 (28.6%) with VNT	LSM: Any 21.1 kPa, AUC 0.85 VNT/large 29.3 kPa AUC 0.76	LSM: Any Sens. 84%, Spec. 71% VNT Sens. 81%, Spec. 61%	*•* Parameters: Prothrombin index *•* VNT: large EV
Castéra, *J Hepatol*, 2009 [21]	Prospective Etiology: HCV	*n* = 70 patients with cirrhosis analyzed *n* = 25 with EV *n* = 13 with VNT	LSM: * Any 13.9 kPa (Kazemi 2006) 17.6 kPa (Vizutti 2007) 21.5 kPa, AUC 0.96 * VNT 19 kPa (Kazemi 2006) 21.5 kPa, AUC 0.87 30 kPa, AUC 0.87	LSM: *Any: Cutoff 13.9 kPa: Sens. 96%, Spec. 39%, PPV 49%, NPV 94% Cutoff 17.6 kPa: Sens. 84%, Spec. 61%, PPV 57%, NPV 86% Cutoff 21.5 kPa: Sens. 76%, Spec. 78%, PPV 68%, NPV 84% *VNT: Cutoff 19.0 kPa: Sens. 85%, Spec. 62%, PPV 35%, NPV 94% Cutoff 21.5 kPa: Sens. 85%, Spec. 68%, PPV 39%, NPV 95% Cutoff 30.5 kPa: Sens. 77%, Spec. 5%, PPV 56%, NPV 94%10	*•* Parameters: PLT, FibroTest, prothrombin index, AST/ALT ratio, APRI, Lok index
Kim, *Am J Gastroenterol*, 2010 [22]	Prospective Etiology: HBV	*n* = 391 *n* = 184 (47%) with EV *n* = 133 (34%) with VNT	LSM: n/a LSPS: Any: n/a VNT 3.5, AUC 0.95	LSM: n/a LSPS: VNT: Cutoff 3.5: Sens. 88%, Spec. 91%, PPV 82%, NPV 94% VNT: Cutoff 5.5: Sens. 72%, Spec. 98% PPV 94%, NPV 88%	• Diagnostic accuracy varies with severity of cirrhosis: LPS: AUC 0.94 (Child-Pugh A), AUC 0.88 (Child-Pugh B/C). • Included cirrhosis Child-Pugh A-C, training cohort and validation cohort
Nguyen-Khac, *Alcohol Clin Exp Res*, 2010 [23]	Prospective Mixed etiologies	*n* = 183 patients analyzed *n* = 41 (22%) with large EV/VNT	LSM: VNT 48 kPa; AUC 0.75 VNT (ALD) 47.2 kPa, AUC 0.77 VNT (viral) 19.8 kPa, AUC 0.73	LSM: VNT: Sens. 73%, Spec. 73%, PPV 44%, NPV 90% VNT (ALD): Sens. 85%, Spec. 64%, PPV 44%, NPV 93% VNT (viral): Sens. 89%, Spec. 55%, PPV 27%, NPV 96%	
Stefanescu, *J Gastroenterol Hepatol*, 2011 [24]	Prospective Etiology: ALD and/or HCV or healthy controls	*n* = 137 with cirrhosis analyzed *n* = 116 (85%) with EV *n* = 60 (44%) with VNT	LSM: Any 28 kPa, AUC 0.75 SSM: Any 46.4 kPa, AUC 0.78 LSM + SSM: Any: LSM 19 kPa, SSM 55 kPa	LSM: Any: Sens. 74%, Spec. 64%, PPV 92%, NPV 31% SSM: Any: Sens. 84%, Spec. 71%, PPV 94%, NPV 46% LSM (19 kPa) + SSM (55 kPa): Any: Sens. 93%, Spec. 40%, PPV 95%, NPV 33%	*•* Parameters: PLT/spleen size ratio; LSM × SSM *•* VNT: cutoffs n/a
Stefanescu, *J Gastrointest Liver Dis*, 2011 [25]	Prospective Etiology: ALD and/or HCV	*n* = 231 patients analyzed *n* = 157 (68%) with EV *n* = 68 (30%) with VNT	LSM: Any 19 kPa, AUC 0.66 VNT 38 kPa, AUC 0.69	LSM: Any: Sens. 84%, Spec. 32%, PPV 72%, NPV 49% VNT: Sens. 56%, Spec. 75%, PPV 47%, NPV 81%	*•* Included cirrhosis *•* Excluded decompensated cirrhosis, co-infection with viral hepatitis *•* Parameters: APRI, Forns Index, Lok Score, FIB4 *•* VNT: grades 2–3 EV
Chen, *J Gastroenterol Hepatol*, 2012 [26]	Prospective Etiology: HBV	*n* = 222 patients analyzed *n* = 96 (43%) with EV *n* = 82 (40%) with VNT	LSM: VNT 17.1 kPa, AUC 0.73 (all) VNT 36.1 kPa, AUC 0.92 (ALT > 5 × ULN) VNT 7.9 kPa, AUC 0.79 (Child-Pugh A, rule out EV) VNT 34.6 kPa (Child-Pugh A, rule in EV) LSPS: VNT Cutoff 3.5, cutoff 5.5; AUC 0.81	LSM: VNT: Cutoff 17.1 kPa: Sens. 90%, Spec. 44%, PPV 47%, NPV 88% VNT: Cutoff 36.1 kPa: PPV 73%, NPV 100% VNT: Cutoff 7.9 kPa: Sens. 97%, NPV 95% VNT: Cutoff 34.6 kPa: Spec. 94%, PPV 73% LSPS: VNT: Cutoff 3.5: Sens. 78%, NPV 86% (exclusion) VNT: Cutoff 5.5: Spec. 90%, PPV 76% (inclusion)	*•* Included cirrhosis *•* Excluded history of variceal bleeding, NSBB therapy, TIPS *•* VNT: medium or large EV, or small with RSS, or decompensated cirrhosis *•* Parameters: USLS; LSM × ALT, LSM × Child-Pugh, LSPS, age-PLT-AST/ALT ratio, PLT/spleen diameter ratio
Wang, *J Gastroenterol Hepatol*, 2012 [27]	Prospective Etiology: HBV	*n* = 126 patients analyzed *n* = 48 (38%) with EV *n* = 13 (10%) with VNT	LSM: Any 12.0 kPa, AUC 0.79 VNT 21.0 kPa, AUC 0.87	LSM: Any: Sens. 67%, Spec. 77%, PPV 64%, NPV 79% VNT: Sens. 77%, Spec. 87%, PPV 40%, NPV 97%	*•* Parameters: *•* APRI, PLT, AST/ALT ratio
Colecchia, *Gastroenterology*, 2012 [28]	Prospective Etiology: HCV	*n* = 100 patients analyzed *n* = 53 (53%)with EV *n* = 26 (26%) with VNT	LSM (AUC 0.90): Any: cutoff 16.4 kPa Any: cutoff 25.0 kPa SSM (AUC 0.94): Any: cutoff 41.3 kPa Any: cutoff 55.0 kPa LSPS (AUC 0.91) Any: cutoff 1.32 Any: cutoff 3.83	LSM: Any: cutoff 16.4 kPa; Sens. 96%, Spec. 60% (rule out) Any: cutoff 25.0 kPa, Sens. 57%, Spec. 98% (rule in) SSM: Any: cutoff 41.3 kPa, Sens. 98%, Spec. 66% (rule out) Any: cutoff 55.0 kPa, Sens. 72%, Spec. 96% (rule in) LSPS: Any: cutoff 1.32: Sens. 98%, Spec. 64% (rule out) Any: cutoff 3.83: Sens. 60%, Spec. 98% (rule in)	• LSM and SSM for detection of HVPG > 12 mmHg and EV • Parameters: • LSPS, PLT/spleen size
Calvaruso, *J Viral Hepat*, 2013 [29]	Prospective Etiology: HCV	*n* = 96 patients analyzed *n* = 54 (56.3%) with EV *n* = 26 (27.1%) with VNT	LSM: Any 17.0 kPa, AUC 0.71 VNT 19.0 kPa, AUC 0.71 Modified SSM (0-150 kPa): Any 50.0 kPa, AUC 0.70 VNT 54.0 kPa, AUC 0.82	LSM: Any: Sens. 71%, Spec. 57%, PPV 67%, NPV 62% VNT: Sens. 72% Spec. 55%, PPV 38%, NPV 84% Modified SSM: Any: Sens. 65%, Spec. 61%, PPV 69%, NPV 57% VNT: Sens. 80%, Spec. 70%, PPV 47%, NPV 90%	• Modified SSM: values 0–150 kPa • Parameters: Gender, age, AST, ALT, PLT, AST/ALT ratio, APRI, spleen diameter
Shi, *Liver Int*, 2013 [30]	Meta-analysis All etiologies, with sub-analyses for viral etiology (HCV + HBV)	*n* = 3644 patients analyzed *n* = 1786? (49.0%) with EV *n* = 1166? (32.0%) with VNT	LSM (pooled): Any: Cutoffs 15.1–28.0 kPa; AUC 0.84 VNT: Cutoffs: 17.8–48.0 kPa; AUC 0.78	LSM (pooled): Any: Cutoffs 15.1–28.0 kPa: Sens. 87%, Spec. 53%, PPV 79%, NPV 64% VNT: Cutoffs: 17.8–48.0 kPa: Sens. 86%, Spec. 59%,, PPV 79%, NPV 66%	
Sharma, *Am J Gastroenterol*, 2013 [31]	Prospective Mixed etiologies	*n* = 174 patients analyzed *n* = 124 patients with EV *n* = 78 with large EV/VNT	LSM: Any 27.3 kPa, AUC 0.91 VNT: n/a SSM: Any 40.8 kPa, AUC 0.90 LSPS: Any 3.09, AUC 0.87	LSM: Any: Sens. 91%, Spec. 72%, PPV 89%, NPV 76% SSM: Any: Sens. 94%, Spec. 76%, PPV 91%, NPV 84% LSPS: Any: Sens. 89%, Spec. 76%, PPV and NPV not reported	• Only LS und SS independently associated with presence of EV; • SSM: can differentiate between large and small EV • LSM: Cannot differentiate between large and small EV • Included cirrhosis • Excluded decompensated cirrhosis, ACLF, active alcohol abuse • Parameters: LSPS, PLT/spleen diameter ratio
Binţinţan, *Med Ultrason*, 2015 [32]	Prospective Etiology: viral and/or ALD	*n* = 60 patients with cirrhosis *n* = 47 (78%) with EV *n* = 32 (53%) with VNT	LSM: Any 15 kPa, AUC 0.96 VNT 28.8 kPa, AUC 0.90	LSM: Any: Sens. 95%, Spec. 100%, PPV 100%, NPV 86% VNT: Sens. 87%, Spec. 83%, PPV 84%, NPV 86%	*•* Parameters: hemodynamic liver index, portal vascular resistance, spleno-portal index
Hu, *Ultrasound Med Biol*, 2015 [33]	Prospective Etiology: viral (HBV, HCV)	*n* = 200 patients analyzed *n* = 110 (55%) with EV *n* = 69 (35%) with large EV	LSM: Any 20.3 kPa, AUC 0.84 VNT 25.6 kPa, AUC 0.86	LSM: Any: Sens. 84%, Spec. 73%, PPV 72%, NPV 91% VNT: Sens. 86%, Spec. 72%, PPV 79%, NPV 81%	*•* VNT: grade 2 or 3 EV *•* Parameters: LSM x PLT (no detailed description available)
Marot, *Liver Int*, 2017 [34]	Meta-analysis Mixed etiologies	*n* = 3364 *n* = (49.0%) with EV *n* = (32.0%) with VNT	LSM: Any 20 kPa* LSM and PLT (150 G/L): VNT 20 kPa × PLT 150 G/L*	LSM and PLT (150 G/L): Any: Sens. 89%, Spec. 38%, PPV 43%, NPV 86% VNT: Sens. 93%, Spec. 30%, PPV 14%, NPV 97%	• Focus on risk of bleeding rather than finding cutoffs for prediction of EV • VNT: variable definition between studies
Pu, *J Gastroenterol*, 2017 [35]	Meta-analysis Mixed etiologies	*n* = 2697 patients analyzed Patients with EV: n/a	LSM (pooled): Any 20 kPa, AUC 0.83 VNT 30 kPa, AUC 0.83	LSM: Any (pooled): Sens. 84%, Spec. 62%, Any: Cutoff 20 kPa: Sens. 83%, Spec. 68%, PPV: n/a, NPV: n/a VNT (pooled): Sens. 78%, Spec. 76%, VNT: Cutoff 30 kPa: Sens. 73%, Spec. 74%, PPV: n/a, NPV: n/a	*•* VNT: large EV
Llop, J *Gastroenterol Hepatol*, 2017 [36]	Retrospective analysis of prospective data Mixed etiology	*n* = 161 patients analyzed *n* = 25 (15.5%) with EV VNT: n/a	LSM Any 20.0 kPa* LSM + PLT:* Any 20.0 kPa x PLT 150 G/L* LSPS: Any: cutoff 3.21;	LSM: Any: Cutoff 20 kPa*: Sens. 76%, Spec. 71%, PPV 32%, NPV 94% LSM and PLT (150 G/L)*: Any: Cutoff 20 kPa*: Sens. 88%, Spec. 38%, PPV 21%, NPV 94% LSPS: Any: Sens. 36%, Spec. 84%, PPV 30%, NPV 88%	*•* TE is the best single method to predict EV; LSPS had high and Baveno VI criteria low risk of misclassifying patients with EV *•* Included LSM >10 kPa *•* Excluded decompensated cirrhosis *•* Parameters: PLT, spleen diameter, LSPS, variceal risk index *•* Augustin algorithm, Baveno VI *•* AUC was calculated but not reported
Wong, *Liver Int*, 2018 [37]	Prospective	LSM: Total *n* = 274/548 *n* = 51 (18.6%) with EV *n* = 11 (4.0%) with VNT	LSM: Any 12.5 kPa* VNT 20.0 kPa × PLT 150 G/L* SSM: VNT 41.3 kPa	LSM: Any: Cutoff 20 kPa*: Sens 96%, Spec. 91%, PPV 26%, NPV 47% VNT: Cutoff 12.5 kPa: Sens. 98%, NPV: 94% VNT: Cutoff 20 kPa*: Sens. 91%, Spec. 18.1%, PPV 10%, NPV 96% SSM: Any 98%, NPV 94%	*•* LSM (12.5 kPa) × SSM (41.3 kPa)
Manatsathit, *J Gastroenterol Hepatol*, 2018 [38]	Meta-analysis Mixed etiologies	Any EV: *n* = 1681/3001 (56% of LSM) and *n* = 968/1911 (51% of SSM) with EV VNT: *n* = 1466/4337 (34% of LSM) and *n* = 383/1119 (34% of SSM) with VNT	LSM (pooled): Any: AUC 0.82 VNT: AUC 0.83 SSM (pooled): Any: AUC 0.90 VNT: AUC 0.81 LSPS (pooled): Any: AUC 0.85 VNT: AUC 0.86	LSM (pooled): Sens. 84%, Spec. 64% Any: Sens. 85%, Spec. 64% VNT: Sens. 85%, Spec. 63% SSM (pooled): Sens. 91% Spec. 66% Any: Sens. 90%, Spec. 73% VNT: Sens. 87%, Spec. 52% LSPS (pooled): Any: Sens. 91%, Spec. 67% VNT: Sens. 82%, Spec. 87%	*•* Sub-analyses regarding method (TE, ARFI, others), etiology (ALD vs viral), ethnicity (Asian vs Western), and cACLD vs decompensated cirrhosis *•* No cutoffs calculated!

*ALD*, alcoholic liver disease; *ACLF*, acute on chronic liver failure; *APRI*, AST to PLT ratio index; *ARFI*, acoustic radiation force impulse; *AUC*, area under the (receiver operating) curve; *cACLD*, compensated advanced chronic liver disease; *EV*, esophageal varices; *F*, fibrosis stage; *FIB4*, fibrosis 4 (score); *kPa*, kilopascal (unit); *LS(M)*, liver stiffness (measurement); *LSPS*, liver-stiffness-to-spleen-diameter-to-platelet-ratio score; *NPV*, negative predictive value; *PLT*, platelet count; *PPV*, positive predictive value; *RSS*, red spot sign; *SS(M)*, spleen stiffness (measurement); *TE*, transient elastography; *TIPS*, transjugular intrahepatic portosystemic shunt; *ULN*, upper limit of normal; *USLS*, ultrasound and liver stiffness score; *VNT*, varices in need of treatment

The asterisk * refers to validation data for the cutoff recommend by the Baveno VI consensus conference

Multiple studies have evaluated the diagnostic performance of TE alone and/or in combination with other markers (most commonly platelet count or spleen size) to predict the presence/absence of EV and VNT (see Table 1).

Regarding the detection of the presence of varices of any size using TE alone, the proposed cutoffs vary between 13.9 kPa [18] and 29.7 kPa [40]. The corresponding positive predictive values (PPV) ranged from 26% [37] to 100% [32] while the negative predictive values (NPV) varied between 31% [24] and 94% [36], depending on whether ruling in or ruling out EV was the main aim of the study. TE cutoffs for predicting VNT were usually higher, ranging from 19.0 kPa [18] to 64.5 kPa [41] with corresponding PPV of 25–90% [19, 41] and NPV of 55–100% [19, 26, 42] respectively.

By applying the Baveno VI criteria to rule in or rule out the presence of small varices or the “extended Baveno VI criteria” by Augustin et al. [43], small varices would be missed in a considerable number of patients. Only few studies assessed the performance of the Baveno VI criteria to screen for any EV and found a considerably low specificity of 38% and PPV of 21–43%, but reasonable sensitivity of 88% and NPV of 86% [34, 36] which is still inferior to the NPV of 97–100% reported for VNT [36, 44, 45].

Using LSM, low cutoffs, such as the one proposed by Kazemi et al. [18] at 13.9 kPa and validated by Castera et al. in 2009 [21], appear to be more suitable for ruling out small varices with a sensitivity of 92–96% and a NPV of 85–94% for varices of all sizes. In a small study including 60 cirrhotic patients with viral hepatitis and/or alcoholic liver disease, Bintintan et al. [32] reported a sensitivity of 95%, a specificity of 100%, a PPV of 100%, and a NPV of 86% using a LSM cutoff at 15 kPa for screening for varices of any size. The only other study reaching similar values with regard to accuracy was performed by Stefanescu et al., who used a combination of LSM (cutoff 19 kPa) and spleen stiffness (SSM, cutoff 55 kPa) to rule in varices of any size with a sensibility of 93% and a PPV of 95% [24].

In summary, LSM using TE in combination with platelet count (i.e., Baveno VI criteria) and/or other non-invasive markers of portal hypertension can be used for ruling out VNT, and thus, avoid unnecessary screening endoscopies in settings where low-risk varices are left untreated. Whether yearly re-evaluation of TE and platelet count is feasible for clinical surveillance and which changes of LSM or SSM or platelet count should call for endoscopic screening must be answered in future studies. As of now, we advise performing endoscopy at any given relevant increase in LSM/SSM or decrease in platelet count as advised by the Baveno VI consensus conference in 2015 [3•]. Importantly, the Baveno VI criteria are not sufficient to rule out small varices/any EV, in clinical practice. Thus, at present, endoscopy is essential if our recommendation to implement medical treatment in all patients with low-risk varices is prefered.

### Natural History of Small Varices and Incidence of Bleeding

An important prospective “natural history” study evaluated the incidence of small varices in 113 patients with cirrhosis of different etiologies without varices at baseline endoscopy and found an incidence rate of 5% (0.8–8.2%) and 28% (21.0–35.0%) at 1 and 3 years, respectively [9]. Interestingly, only 2% (0.1–4.1%) of patients without varices at first endoscopy bled within 2 years. Among 93 with small EV at baseline, the rate of progression to large EV was 12% (5.6–18.4%) and 31% (21.2–40.8%) at 1 and 3 years, respectively. Alcoholic etiology of liver disease, advanced liver dysfunction (Child-Pugh B/C), and red spots signs were all risk factors for progression from small to large varices [9]. Bleeding at 2 years occurred in 12% (5.2–18.8%) and the presence of red spot signs at first endoscopy was identified as independent risk factors for variceal bleeding in patients with small varices at baseline [9].

### Pathophysiological Considerations Supporting Current Recommendations for the Management of Portal Hypertension/Patients with Small Varices

Liver fibrosis/cirrhosis increases intrahepatic vascular resistance, and thus, portal pressure [46]. Increases in portal pressure, that is indirectly assessed by the measurement of HVPG, to values of ≥ 10 mmHg denote CSPH [47, 48•]. CSPH is associated with a substantially increased risk for developing EV and/or decompensating events, such as variceal bleeding as well as ascites and its complications. The diagnosis of CSPH is established by HVPG measurement; however, the presence of collaterals such as EV also indicates CSPH. Thus, in clinical practice, CSPH is most commonly diagnosed by endoscopy (e.g., the finding of small varices), as HVPG measurement is mostly restricted to academic centers.

Since portal hypertension drives the development of EV and hepatic decompensation, lowering HVPG by NSBB treatment provides a clinically relevant benefit [47, 48•]. However, a landmark study by Groszmann and Garcia-Tsao et al. [49], in which patients with cirrhosis and portal hypertension (defined by a hepatic venous pressure gradient [HVPG] ≥ 6 mmHg) but without varices were randomized to timolol or placebo, demonstrated that NSBB therapy is not generally effective to prevent the occurrence of EV [49]. Although this might seem contradictory, the increasing knowledge on the pathophysiology of portal hypertension and EV development facilitates the interpretation of the findings of this study [50]. CSPH usually develops prior to the occurrence of small EV, in turn, almost every patient with EV has already developed CSPH. While the initial trigger for CSPH development is an increase in intrahepatic vascular resistance, further increases in portal pressure/HVPG are driven by splanchnic vasodilation and hyperdynamic circulation. Since the latter two mechanisms are the targets of conventional NSBB (propranolol, nadolol, or timolol), it becomes evident that most patients without EV, who have a low likelihood of CSPH, do not benefit from NSBB [51••]. These pathophysiological considerations are also reflected by the “window hypothesis” by Krag et al. [52]. In turn, patients with CSPH who have not yet developed EV might also benefit from NSBB therapy if HVPG can be decreased by > 10% or to absolute values < 10 mmHg [49].

### Impact of Etiological Treatment on Small Varices

The importance of curing the underlying liver disease was most evident from studies assessing the clinical course of compensated cirrhotic patients with viral hepatitis B and C after successful antiviral treatment in comparison to patients with virological treatment failure.

Lee et al. have reported two cases of patients with HCV-related cirrhosis, who showed complete regression of their esophageal varices (and splenomegaly) 3 and 8 years after sustained virological response [53]. A prospective observational study assessed the course of EV in patients with versus without sustained virological response (SVR) to interferon/ribavirin treatment in patients with compensated HCV-related cirrhosis [54]. Sixty-two patients with SVR and 65 patients without SVR were endoscopically followed for a median of about 5 years (68 months vs. 57 months, respectively). Significantly less patients with SVR developed de novo EV (3.5% vs. 15.1% without SVR), but SVR only non-significantly decreased the progression from small to large EV. Since some patients with SVR developed de novo EV or had progression to large EV, the authors concluded that continued endoscopical surveillance for EV is still necessary in patients with HCV cirrhosis despite SVR [9].

Another case of complete regression of EV after suppression of hepatitis B virus (HBV) replication with entecavir has been reported by Jwa et al. [55]. The beneficial impact of long-term nucleos(t)ide analogues (NA) treatment on patients with small EV has been elegantly demonstrated in a prospective, 12-year multicenter study [56••]. Among the 107 HBeAg(−) patients with compensated cirrhosis, 27 patients had small EV at baseline. Long-term suppression of HBV replication by NA resulted in regression of small EV in most cases (83% cumulative incidence of regression over 12 years, 18/27) and no single patient experienced variceal bleeding. The risk of progression from small to large EV was negligible and occurred almost exclusively in patients with virological breakthrough under NA treatment [56••].

In contrast to patients with viral hepatitis, there are currently no specific therapies approved for alcoholic liver disease (ALD) or non-alcoholic steatohepatitis (NASH). In ALD, strict alcohol abstinence and supervised lifestyle modifications represent critical elements of patient management [57, 58]. Up to date, there are no studies that specifically focus on the impact of alcohol consumption/cessation on the progression of small varices. However, it is well-established that even moderate alcohol consumption worsens portal hypertension and thereby most likely affects the formation and growth of varices. After alcohol cessation, an immediate decrease of HVPG can be observed, which might induce regression of varices [59]. Moreover, Villanueva et al. showed that alcohol cessation improves the hemodynamic response in secondary prophylaxis [60]. In addition, durable alcohol abstinence results in lower re-bleeding rates [61] and decreased long-term mortality after acute variceal bleeding [62]. Negative effects of ongoing alcohol consumption seem to result from increased intrahepatic resistance, due to hepatic inflammation, characterized by parenchymal lesions (hepatocellular ballooning) that are frequently found in active drinkers [63]. In conclusion, alcohol abstinence reduces portal pressure and improves the clinical couse of cirrhotic patients and should therefore be advised to all patients with small varices.

While NASH has become a highly prevalent liver disease [64], there are currently no approved pharmacological therapies for patients with NASH. The “Million Women Study,” performed in the UK, suggested that up to 17% of incident cases of cirrhosis can be directly linked to obesity [65]. Obesity in combination with cirrhosis increases risk of hepatic decompensation and negatively impacts portal hypertension [66]. The pathophysiological mechanisms are still unknown and certainly multifactorial. However, it seems that a persistent proinflammatory state triggers a fibrogenic and angiogenic response in the liver, thereby increasing intrahepatic resistance. In NASH, an increase in portal pressure has been reported in the absence of significant fibrosis [67] with hepatocellular ballooning due to lipotoxicity and/or only mild perisinusoidal fibrosis [9]. Lifestyle interventions remain the key determinants of NASH progression/regression: Weight loss is associated with decreases in HVPG irrespectively of etiology [68••]. Importantly, beneficial effects of weight loss may be observed even in the absence of changes in Child-Pugh or MELD score [66, 68••]. In selected cases, bariatric surgery might be beneficial in NASH patients [69].

### Treatment of Small Varices with Non-selective Betablockers (Table 2)

Based on a broad body of evidence, current guidelines recommend either NSBB or EBL for primary prophylaxis of variceal bleeding in patients with medium to large varices. Moreover, NSBB are considered as the mainstay of the combination therapy with EBL therapy in secondary prophylaxis [5, 79]. However, in patients with small varices who have not bled, the situation is less clear [3•, 6, 80].

**Table 2:** Proportion of patients with small varices in studies evaluating non-selective betablocker therapy

Author, journal, year	Design	*N* patients overall *N* with small EV (%)	NSBB (dose)	HVPG measurement	Main conclusions
The PROVA Study Group, *Hepatology*, 1991 [70]	RCT	286/166 (58%)	Propranolol (160–400 mg/day)	No	• Small EV had a considerable risk of bleeding • The incidence of variceal hemorrhage and overall mortality was not significantly different between patients receiving NSBB, sclerotherapy or combination therapy
Calès, *Eur J Gastroenterol Hepatol*, 1999{Cales, 1999 #1480}	RCT	206/127 (62%)	Propranolol (160 mg)	No	• NSBB therapy did neither prevent occurrence/growth of EV or variceal bleeding and did not reduce mortality in patients without/with small EV
Merkel, *Hepatology*, 2000 [71]	RCT	146/6 (4.1%)	Nadolol (40–160 mg/day)	No	• NSBB plus ISMN was more effective than NSBB alone in the long-term prophylaxis of first variceal bleeding
Merkel, *Hepatology*, 2000 [72]	RCT	49/2 (4.1%)	Nadolol (40–80 mg/day)	Yes (all)	• HVPG reponse was the best predictor of efficacy in patients receiving NSBB or NSBB plus ISMN for primary prophylaxis
Abraczinskas, *Hepatology*, 2001 [73]	RCT	49/32 (65.3%)	Propranolol (dose not specified)	No	• NSBB therapy in small and large EV is effective in preventing of first variceal bleeding • After discontinuation of NSBB therapy, the risk of variceal bleeding persisted
Merkel, *Gastroenterology*, 2004 [7]	RCT	161 (100%)	Nadolol (mean dose 62 ± 25 mg/day)	Yes (11.8%)	• Primary prophylaxis with NSBB should be considered in patients with small EV • NSBB delay the growth of small EV
Turnes, *Am J Gastroenterol*, 2006 [74]	RCT	71/4 (6.6%)	Propranolol (54 ± 14 to 79 ± 12 mg/day)	Yes (all)	• Positive impact of HVPG response in the setting of primary prophylaxis • Insufficient data on patients with small EV
Reiberger, *Gut*, 2013 [75]	Non-randomized clinical trial	104/41 (39.4%)	Propranolol (80–160 mg/day) Carvedilol (6.25–50 mg/day)	Yes (all)	• Carvedilol induces HVPG response in a considerable proportion of patients with propranolol non-response • Patients with small EV were included and also benefited from hemodynamic response to carvedilol
Sarin, *Hepatol Int,* 2013 [8]	RCT	150 (100%)	Propranolol (40 mg/day followed by dose titration)	Yes (66%)	• NSBB therapy did neither prevent growth of EV or variceal bleeding and did not reduce mortality in patients with small EV
Je, *Clin Mol Hepatol*, 2014 [76]	Retrospective study	504/92 (18.3%)	Propanolol (20 mg/day followed by dose titration)	No	• NSBB plus EBL was more effective than NSBB alone in primary prophylaxis • However, EBL was performed only in patients with large EV
Bhardwaj, *Gut*, 2016 [77]	RCT	70 (100%)	Carvedilol (mean dose 12 ± 1.67 mg/day)	Yes (all)	• Reduction of progression to large EV
Kim, *Dig Dis Sci*, 2016 [78]	Retrospective study	898/775 (86.3%)	48.6% of 898 patients were on NSBB therapy	No	• Variceal bleeding was a risk factor for mortality in patients with hepatocellular carcinoma
Pfisterer, *Aliment Pharmacol Ther*, 2018 [1]	Retrospective study	Primary prophylaxis: 281/48 (17.1%)	Propranolol (median dose 40 mg/day) Carvedilol (median dose 12.5 mg/day)	No	• Addition of EBL to NSBB therapy did not further reduce the risk of first variceal bleeding or mortality • Patients receiving HVPG-guided primary prophylaxis (including patients with small EV) tended to have a better prognosis than patients receiving non-HVPG-guided NSBB therapy or combination therapy

*EBL*, endoscopic band ligation; *EV*, esophageal varices; *HVPG*, hepatic venous pressure gradient; *NSBB*, non-selective betablocker; RCT, randomized controlled trial

In general, there is no conclusive evidence that NSBB treatment reduces the risk of variceal bleeding or mortality in patients with low-risk varices who have not bled [5]. However, most trials were not sufficiently powered to detect favorable effects on these endpoints, which occur less commonly in this group of patients [6]. This is the reason why well-designed studies on clinically meaningful surrogate endpoints (e.g., variceal growth) are particularly relevant in this setting.

While a randomized controlled trial (RCT) by Merkel et al. [7] demonstrated that conventional NSBB (nadolol) therapy is effective in preventing the progression from small to large varices in patients who have not bled, another RCT by Sarin and co-workers [8] using propranolol reported no effect. In a recently published randomized RCT by Bhardwaj and colleagues [77], a lower proportion of patients assigned to the carvedilol group progressed from small to large varices as compared to the placebo group. Carvedilol has been shown to be more potent in decreasing portal pressure due to its additional anti-α1-adrenergic activity [81]. In a recent meta-analysis [82], the mean relative HVPG reduction was 22% for carvedilol and 16% for propranolol, resulting in a weighed mean difference of 7% in favor of carvedilol. However, owing to its anti-α1-adrenergic activity [81], carvedilol might also lead to more pronounced decreases in systemic arterial pressure, when compared to conventional NSBB, which limits its use in patients with severe or refractory ascites [5, 79•].

We recently conducted a meta-analysis of RCT on NSBB treatment restricted to patients with small varices, also including the new study by Bhardwaj and co-workers [77]. Interestingly, in our meta-analysis [83], there was a trend towards an amelioration of the progression from small to large varices among NSBB-treated patients in the fixed effects model (odds ratio [OR] [95% confidence interval (95% CI)] 0.73 [0.5–1.06]; risk ratio [RR] [95% CI] 0.78 [0.58–1.05]). However, the random effects model might have been more accurate due to study heterogeneity. In this model, the 95% CI was substantially wider (OR [95% CI] 0.76 [0.25 to 2.29]; RR [95% CI] 0.82 [0.36 to 1.87]). Thus, the findings of our meta-analysis [83] plead for further studies to confirm the effectiveness of NSBB therapy (especially carvedilol) for preventing variceal growth.

### The Role of Hepatic Venous Pressure Gradient in Guiding Treatment Decisions in Patients with Small Varices

Considering varices of all size (i.e., also medium to large varices), NSBB treatment reduces the 2-year risk of first variceal bleeding from 25 to 15%, resulting in a number needed to treat [NNT] of 10 [84]. In the group of patients with small varices, however, the NNT for preventing variceal bleeding might be considerably higher (about 20), underlining the importance of identifying patients who are most likely to benefit. To date, HVPG response is the only established surrogate for the effectiveness of NSBB therapy. Due to the need for two separate HVPG measurements, the evaluation of “chronic” HVPG response to NSBB treatment is very resource-intensive. In contrast, the assessment of “acute” HVPG response to i.v. propranolol is performed in a single session, and thus, provides a valuable alternative [85, 86]. For primary prophylaxis, the Baveno VI faculty recently unified the definition of HVPG response by using the same criteria (HVPG decrease ≥ 10% or to a value of ≤ 12 mmHg) for “acute” and “chronic” assessments [3•]. Although most of the evidence for the impact of a decrease in HVPG on hepatic decompensation [87] and mortality [88] is derived from studies comprising patients with medium-large varices, there are also some studies focusing on patients with less advanced disease. In a landmark study [49] assigning patients with portal hypertension who had not developed varices (pre-primary prophylaxis) to timolol or placebo, patients who had a relative HVPG decrease of > 10% after 1 year showed a reduced incidence of the composite primary endpoint (development of varices or variceal bleeding) [49]. Moreover, a recent RCT evaluated the impact of HVPG-guided therapy on hepatic decompensation in patients with clinically significant portal hypertension (CSPH; pre-primary prophylaxis [44%] or small varices without red spot signs [56%]). Two hundred one patients were randomized to NSBB therapy (propranolol or carvedilol in patients with and without “acute” HVPG response to i.v. propranolol, respectively) or placebo. Interestingly, the rates of hepatic decompensation were 16% and 27% in the NSBB and placebo group, respectively. Thus, HVPG-guided therapy with propranolol/carvedilol substantially reduced the risk of hepatic decompensation (hazard ratio [95% CI] 0.51 [0.58 to 1.05]), primarily by decreasing the incidence of ascites [89••], which is the most frequent first decompensating event [90].

Since nearly all patients with cirrhosis and small varices have CSPH, the findings of the aforementioned study [89••], as well as the potential effect of NSBB treatment on the progression of varices, provide a good rationale for HVPG-guided NSBB therapy in patients with small varices. This approach allows to identify patients who are likely to benefit from propranolol, while hemodynamic non-responders can be treated with carvedilol, which still achieves HVPG response in a significant proportion of these patients [69]. Moreover, information on HVPG response might facilitate treatment individualization in situations in which NSBB might have a less favorable safety profile [5, 79•], e.g., patients with refractory ascites [91], spontaneous bacterial peritonitis [92], or severe alcoholic hepatitis [93]. If HVPG measurement is not available, carvedilol 12.5 mg q.d. might be the NSBB of choice in patients without severe ascites.

### Our Personal Recommendations for Screening and Management of Small Varices (Fig. 1)

Here, we want to provide the reader with recommendations for daily clinical practice, that—in absence of RCT on some specific aspects—may not always be based on high-quality evidence. First, TE cutoffs < 20 kPa applied to patients with cACLD even combined with platelet count > 150 G/L cannot sufficiently rule out the presence of small varices. Accordingly, endoscopy is essential if low-risk varices are intended to be treated with pharmacological therapy. If liver stiffness is measured at ≥ 20 kPa and/or platelet count is < 150 G/L, screening endoscopy must be performed regardless of the treatment strategy. Moreover, the occurrence of hepatic decompensation should prompt endoscopy.

**Fig. 1:**
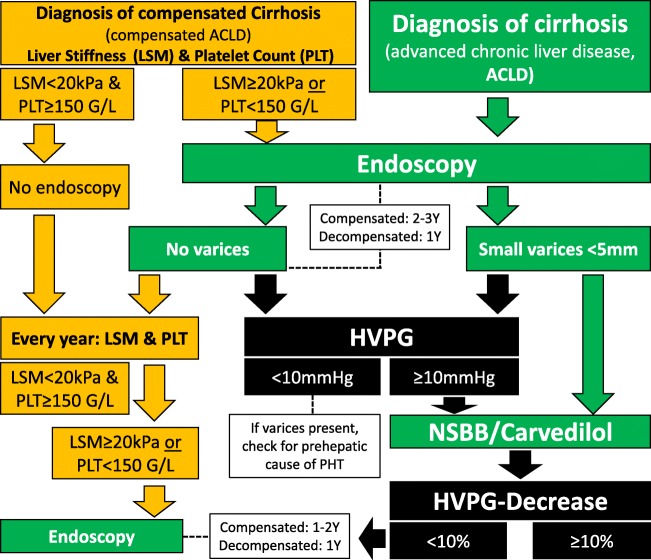
Suggested algorithm for the diagnosis and management of small varices. *The green elements summarize the recommended strategy as supported by previous international guidelines* that recommend screening endoscopy in all patients diagnosed with cirrhosis (i.e., ACLD). If small varices are detected, treatment with conventional NSBB or carvedilol may be started as primary prophylaxis of variceal bleeding. If no varices are detected, screening endoscopy should be repeated every 1–2 years. *The orange elements summarize the Baveno VI recommendations for non-invasive diagnosis of varices* in patients with cirrhosis that were designed to avoid screening endoscopies in patients with low liver stiffness (TE < 20 kPa) and normal platelet counts (PLT ≥ 150 G/L). However, since this strategy will miss a considerable number of patients with small varices (that must not be treated—but should be treated in our opinion), we would still recommend to perform screening endoscopies even in patients with TE ≥ 20 kPa or PLT < 150 G/L if cirrhosis (ACLD) is suspected. *Finally, the black elements indicate advanced diagnostic/therapeutic options that we recommend for optimal management* of patients with small varices: In patients with small varices and even in patients with ACLD without varices (especially in those with TE ≥ 20 kPa or PLT < 150 G/L), HVPG should be measured. If HVPG is measured at ≥ 10 mmHg, the hemodynamic response to NSBB (ideally intravenous testing) or carvedilol should be assessed and hemodynamic responders should be kept on NSBB or carvedilol if a decrease of at least ≥ 10% in HVPG is observed. In hemodynamic non-responders, follow-up endoscopy should be performed every 1–2 years in order to assess the progression to large varices. ACLD, advanced chronic liver disease; cACLD compensated advanced chronic liver disease; HVPG, hepatic venous pressure gradient; LSM, liver stiffness measurement; NSBB, non-selective betablocker; PLT, platelet count; Y, year(s)

Varices should then be classified as absent, small (< 5 mm), or large (≥ 5 mm). Further, presence of red spot signs and Child-Pugh score needs to be evaluated for risk stratification.

If small varices are detected, in addition to etiological therapy, we initiate NSBB therapy due to its potential beneficial effects on variceal growth and the reduced risk of hepatic decompensation. Importantly, NSBB seem to be effective even in patients with small (and large) varices who remain strictly abstinent from alcohol [9, 68••, 69, 73] and in patients who receive effective antiviral therapy [68••]. This would support the treatment of small varices both in patients with ongoing hepatic injury and in patients with effective treatment of the underlying etiology. Thus, we use NSBB in all patients with small varices, independently if the etiological factor is eliminated or not.

Acute HVPG response can be evaluated by administration of i.v. propranolol within a single session, while chronic assessment of HVPG response requires a second HVPG measurement around 3–5 weeks after initiation of conventional NSBB/carvedilol therapy. This HVPG-guided approach allows to identify patients who are likely to benefit from propranolol, while hemodynamic non-responders can be treated with carvedilol, which still achieves chronic HVPG response in a significant proportion of these patients. A decrease in HVPG ≥ 10% or to a value of ≤ 12 mmHg defines NSBB response. If HVPG measurement is not available, we recommend the use of carvedilol 12.5 mg q.d. in patients without severe ascites.

In general, we would also recommend NSBB treatment for patients with CSPH but without EV, since HVPG response may prevent the development of varices as well as decompensating events in compensated patients.

In patients with severe/refractory ascites, arterial blood pressure should be monitored since NSBB might compromise the circulatory reserve. The risk-benefit ratio/dosing of NSBB treatment should be re-evaluated in patients with arterial hypotension, spontaneous bacterial peritonitis, and/or acute kidney injury. In some patients, NSBB therapy might need to be discontinued if arterial hypotension is severe or shock/AKI requiring vasopressors occurs; however, this might be indicative of worse prognosis and these patients should then be evaluated for more aggressive treatment strategies such as TIPS or liver transplantation.

## Conclusions

The main therapeutic focus in cirrhotic patients with small varices (≤ 5 mm in diameter) is the cure of the underlying etiology. The optimal management of small varices should include measurement of HVPG. A pharmacological decrease in HVPG by NSBB therapy ≥ 10% reduces the risk of progression to large varices, first variceal bleeding, and hepatic decompensation. If HVPG is not available, we would recommend carvedilol 12.5 mg once daily for treatment of small varices in patients without severe ascites. Only if small EV are not treated or in hemodynamic non-responders, follow-up endoscopies should be performed in 1–2 yearly intervals considering the activity of liver disease or if hepatic decompensation occurs.

## References

[1] Pfisterer N, Dexheimer C, Fuchs EM, Bucsics T, Schwabl P, Mandorfer M, Gessl I, Sandrieser L, Baumann L, Riedl F, Scheiner B, Pachofszky T, Dolak W, Schrutka-Kölbl C, Ferlitsch A, Schöniger-Hekele M, Peck-Radosavljevic M, Trauner M, Madl C, Reiberger T (2018). Betablockers do not increase efficacy of band ligation in primary prophylaxis but they improve survival in secondary prophylaxis of variceal bleeding. Aliment Pharmacol Ther.

[2] Albillos A, Zamora J, Martinez J (2017). Stratifying risk in the prevention of recurrent variceal hemorrhage: results of an individual patient meta-analysis. Hepatology.

[3] de Franchis R, Baveno VIF (2015). Expanding consensus in portal hypertension: report of the Baveno VI Consensus Workshop: stratifying risk and individualizing care for portal hypertension. J Hepatol.

[4] Garcia-Tsao G, Abraldes JG, Berzigotti A, Bosch J (2017). Portal hypertensive bleeding in cirrhosis: risk stratification, diagnosis, and management: 2016 practice guidance by the American Association for the study of liver diseases. Hepatology.

[5] Mandorfer M, Reiberger T (2017). Beta blockers and cirrhosis, 2016. Dig Liver Dis.

[6] Tripathi D, Stanley AJ, Hayes PC, Patch D, Millson C, Mehrzad H, Austin A, Ferguson JW, Olliff SP, Hudson M, Christie JM, Clinical Services and Standards Committee of the British Society of Gastroenterology (2015). U.K. guidelines on the management of variceal haemorrhage in cirrhotic patients. Gut.

[7] Merkel C, Marin R, Angeli P, Zanella P, Felder M, Bernardinello E, Cavallarin G, Bolognesi M, Donada C, Bellini B, Torboli P, Gatta A, Gruppo Triveneto per l’Ipertensione Portale (2004). A placebo-controlled clinical trial of nadolol in the prophylaxis of growth of small esophageal varices in cirrhosis. Gastroenterology.

[8] Sarin SK, Mishra SR, Sharma P, Sharma BC, Kumar A (2013). Early primary prophylaxis with beta-blockers does not prevent the growth of small esophageal varices in cirrhosis: a randomized controlled trial. Hepatol Int.

[9] Merli M, Nicolini G, Angeloni S, Rinaldi V, de Santis A, Merkel C, Attili AF, Riggio O (2003). Incidence and natural history of small esophageal varices in cirrhotic patients. J Hepatol.

[10] Brick IB, Palmer ED (1964). One thousand cases of portal cirrhosis of the liver. Implications of esophageal varices and their management. Arch Intern Med.

[11] Dagradi AE, Stempien SJ, Owens LK (1966). Bleeding esophagogastric varices. An endoscopic study of 50 cases. Arch Surg.

[12] The general rules for recording endoscopic findings on esophageal varices. Jpn J Surg. 1980;10(1):84–7. 10.1007/BF02468653 7373958

[13] Paquet KJ (1982). Prophylactic endoscopic sclerosing treatment of the esophageal wall in varices -- a prospective controlled randomized trial. Endoscopy.

[14] Reiberger T, Puspok A, Schoder M (2017). Austrian consensus guidelines on the management and treatment of portal hypertension (Billroth III). Wien Klin Wochenschr.

[15] Bosch J, Abraldes JG, Groszmann R (2003). Current management of portal hypertension. J Hepatol.

[16] North Italian Endoscopic Club for the S, Treatment of Esophageal V (1988). Prediction of the first variceal hemorrhage in patients with cirrhosis of the liver and esophageal varices. A prospective multicenter study. N Engl J Med.

[17] Foucher J, Chanteloup E, Vergniol J, Castéra L, le Bail B, Adhoute X, Bertet J, Couzigou P, de Lédinghen V (2006). Diagnosis of cirrhosis by transient elastography (FibroScan): a prospective study. Gut.

[18] Kazemi F, Kettaneh A, N’Kontchou G (2006). Liver stiffness measurement selects patients with cirrhosis at risk of bearing large oesophageal varices. J Hepatol.

[19] Vizzutti F, Arena U, Romanelli RG, Rega L, Foschi M, Colagrande S, Petrarca A, Moscarella S, Belli G, Zignego AL, Marra F, Laffi G, Pinzani M (2007). Liver stiffness measurement predicts severe portal hypertension in patients with HCV-related cirrhosis. Hepatology.

[20] Bureau C, Metivier S, Peron JM (2008). Transient elastography accurately predicts presence of significant portal hypertension in patients with chronic liver disease. Aliment Pharmacol Ther.

[21] Castera L, Le Bail B, Roudot-Thoraval F (2009). Early detection in routine clinical practice of cirrhosis and oesophageal varices in chronic hepatitis C: comparison of transient elastography (FibroScan) with standard laboratory tests and non-invasive scores. J Hepatol.

[22] Kim BK, Han KH, Park JY, Ahn SH, Kim JK, Paik YH, Lee KS, Chon CY, Kim DY (2010). A liver stiffness measurement-based, noninvasive prediction model for high-risk esophageal varices in B-viral liver cirrhosis. Am J Gastroenterol.

[23] Nguyen-Khac E, Saint-Leger P, Tramier B, Coevoet H, Capron D, Dupas JL (2010). Noninvasive diagnosis of large esophageal varices by Fibroscan: strong influence of the cirrhosis etiology. Alcohol Clin Exp Res.

[24] Stefanescu H, Grigorescu M, Lupsor M, Procopet B, Maniu A, Badea R (2011). Spleen stiffness measurement using Fibroscan for the noninvasive assessment of esophageal varices in liver cirrhosis patients. J Gastroenterol Hepatol.

[25] Stefanescu H, Grigorescu M, Lupsor M, Maniu A, Crisan D, Procopet B, Feier D, Badea R (2011). A new and simple algorithm for the noninvasive assessment of esophageal varices in cirrhotic patients using serum fibrosis markers and transient elastography. J Gastrointest Liver Dis: JGLD.

[26] Chen YP, Zhang Q, Dai L, Liang XE, Peng J, Hou JL (2012). Is transient elastography valuable for high-risk esophageal varices prediction in patients with hepatitis-B-related cirrhosis?. J Gastroenterol Hepatol.

[27] Wang JH, Chuah SK, Lu SN, Hung CH, Chen CH, Kee KM, Chang KC, Tai WC, Hu TH (2012). Transient elastography and simple blood markers in the diagnosis of esophageal varices for compensated patients with hepatitis B virus-related cirrhosis. J Gastroenterol Hepatol.

[28] Colecchia A, Montrone L, Scaioli E, Bacchi–Reggiani ML, Colli A, Casazza G, Schiumerini R, Turco L, di Biase AR, Mazzella G, Marzi L, Arena U, Pinzani M, Festi D (2012). Measurement of spleen stiffness to evaluate portal hypertension and the presence of esophageal varices in patients with HCV-related cirrhosis. Gastroenterology.

[29] Calvaruso V, Bronte F, Conte E, Simone F, Craxi A, Di Marco V (2013). Modified spleen stiffness measurement by transient elastography is associated with presence of large oesophageal varices in patients with compensated hepatitis C virus cirrhosis. J Viral Hepat.

[30] Shi KQ, Fan YC, Pan ZZ, Lin XF, Liu WY, Chen YP, Zheng MH (2013). Transient elastography: a meta-analysis of diagnostic accuracy in evaluation of portal hypertension in chronic liver disease. Liver Int.

[31] Sharma P, Kirnake V, Tyagi P, Bansal N, Singla V, Kumar A, Arora A (2013). Spleen stiffness in patients with cirrhosis in predicting esophageal varices. Am J Gastroenterol.

[32] Bintintan A, Chira RI, Bintintan VV, Nagy G, Manzat-Saplacan RM, Lupsor Platon M, Stefanescu H, Duma MM, Valean SD, Mircea PA (2015). Value of hepatic elastography and Doppler indexes for predictions of esophageal varices in liver cirrhosis. Med Ultrason.

[33] Hu Z, Li Y, Li C, Huang C, Ou Z, Guo J, Luo H, Tang X (2015). Using ultrasonic transient elastometry (FibroScan) to predict esophageal varices in patients with viral liver cirrhosis. Ultrasound Med Biol.

[34] Marot A, Trepo E, Doerig C, Schoepfer A, Moreno C, Deltenre P (2017). Liver stiffness and platelet count for identifying patients with compensated liver disease at low risk of variceal bleeding. Liver Int.

[35] Pu K, Shi JH, Wang X, Tang Q, Wang XJ, Tang KL, Long ZQ, Hu XS (2017). Diagnostic accuracy of transient elastography (FibroScan) in detection of esophageal varices in patients with cirrhosis: a meta-analysis. World J Gastroenterol: WJG..

[36] Llop E, Lopez M, de la Revilla J, Fernandez N, Trapero M, Hernandez M, Fernández-Carrillo C, Pons F, Martinez JL, Calleja JL (2017). Validation of noninvasive methods to predict the presence of gastroesophageal varices in a cohort of patients with compensated advanced chronic liver disease. J Gastroenterol Hepatol.

[37] Wong GLH, Kwok R, Hui AJ, Tse YK, Ho KT, Lo AOS, Lam KLY, Chan HCH, Lui RA, Au KHD, Chan HLY, Wong VWS (2018). A new screening strategy for varices by liver and spleen stiffness measurement (LSSM) in cirrhotic patients: a randomized trial. Liver Int.

[38] Manatsathit W, Samant H, Kapur S, Ingviya T, Esmadi M, Wijarnpreecha K, McCashland T (2018). Accuracy of liver stiffness, spleen stiffness, and LS-spleen diameter to platelet ratio score in detection of esophageal varices: systemic review and meta-analysis. J Gastroenterol Hepatol.

[39] Friedrich-Rust M, Ong MF, Martens S (2008). Performance of transient elastography for the staging of liver fibrosis: a meta-analysis. Gastroenterology.

[40] Saad Y, Said M, Idris MO, Rabee A, Zakaria S (2013). Liver stiffness measurement by fibroscan predicts the presence and size of esophageal varices in egyptian patients with HCV related liver cirrhosis. J Clin Diagn Res : JCDR.

[41] Pineda JA, Recio E, Camacho A, Macías J, Almodóvar C, González-Serrano M, Merino D, Tellez F, Ríos MJ, Rivero A, Grupo Andaluz de Hepatitis Vírica (HEPAVIR) de la Sociedad Andaluza de Enfermedades Infecciosas (SAEI) (2009). Liver stiffness as a predictor of esophageal varices requiring therapy in HIV/hepatitis C virus-coinfected patients with cirrhosis. J Acquir Immune Defic Syndr (1999).

[42] Jangouk Parastoo, Turco Laura, De Oliveira Ana, Schepis Filippo, Villa Erica, Garcia-Tsao Guadalupe (2017). Validating, deconstructing and refining Baveno criteria for ruling out high-risk varices in patients with compensated cirrhosis. Liver International.

[43] Augustin S, Pons M, Maurice JB, et al. Expanding the Baveno VI criteria for the screening of varices in patients with compensated advanced chronic liver disease*,* Hepatology (Baltimore MD 2017;66(6):1980–1988. 10.1002/hep.29363 28696510

[44] Colecchia A, Ravaioli F, Marasco G, Colli A, Dajti E, di Biase AR, Bacchi Reggiani ML, Berzigotti A, Pinzani M, Festi D (2018). A combined model based on spleen stiffness measurement and Baveno VI criteria to rule out high-risk varices in advanced chronic liver disease. J Hepatol.

[45] Maurice JB, Brodkin E, Arnold F, Navaratnam A, Paine H, Khawar S, Dhar A, Patch D, O’Beirne J, Mookerjee R, Pinzani M, Tsochatzis E, Westbrook RH (2016). Validation of the Baveno VI criteria to identify low risk cirrhotic patients not requiring endoscopic surveillance for varices. J Hepatol.

[46] Tsochatzis EA, Bosch J, Burroughs AK (2014). Liver cirrhosis. Lancet.

[47] Abraldes JG, Sarlieve P, Tandon P (2014). Measurement of portal pressure. Clin Liver Dis.

[48] La Mura V, Nicolini A, Tosetti G, Primignani M (2015). Cirrhosis and portal hypertension: the importance of risk stratification, the role of hepatic venous pressure gradient measurement. World J Hepatol.

[49] Groszmann RJ, Garcia-Tsao G, Bosch J, Grace ND, Burroughs AK, Planas R, Escorsell A, Garcia-Pagan JC, Patch D, Matloff DS, Gao H, Makuch R, Portal Hypertension Collaborative Group (2005). Beta-blockers to prevent gastroesophageal varices in patients with cirrhosis. N Engl J Med.

[50] Bosch J, Groszmann RJ, Shah VH (2015). Evolution in the understanding of the pathophysiological basis of portal hypertension: how changes in paradigm are leading to successful new treatments. J Hepatol.

[51] •• Villanueva C, Albillos A, Genesca J, et al. Development of hyperdynamic circulation and response to beta-blockers in compensated cirrhosis with portal hypertension. Hepatology. 2015; **Excellent study providing evidence that NSBB therapy is more effective after CSPH has developed**. 10.1002/hep.28264 26422126

[52] Krag A, Wiest R, Albillos A, Gluud LL (2012). The window hypothesis: haemodynamic and non-haemodynamic effects of beta-blockers improve survival of patients with cirrhosis during a window in the disease. Gut.

[53] Lee SJ, Cho YK, Na SY, Choi EK, Boo SJ, Jeong SU, Song HJ, Kim HU, Kim BS, Song BC (2016). Regression of esophageal varices and splenomegaly in two patients with hepatitis-C-related liver cirrhosis after interferon and ribavirin combination therapy. Clin Mol Hepatol.

[54] D’Ambrosio R, Aghemo A, Rumi MG (2011). The course of esophageal varices in patients with hepatitis C cirrhosis responding to interferon/ribavirin therapy. Antivir Ther.

[55] Jwa HY, Cho YK, Choi EK, Kim HU, Song HJ, Na SY, Boo SJ, Jeong SU, Kim BS, Lee BW, Song BC (2016). Regression of esophageal varices during entecavir treatment in patients with hepatitis-B-virus-related liver cirrhosis. Clin Mol Hepatol.

[56] Lampertico P, Invernizzi F, Vigano M (2015). The long-term benefits of nucleos(t)ide analogs in compensated HBV cirrhotic patients with no or small esophageal varices: a 12-year prospective cohort study. J Hepatol.

[57] Osna NA, Donohue TM, Kharbanda KK (2017). Alcoholic liver disease: pathogenesis and current management. Alcohol Res : Curr Rev.

[58] Addolorato G, Mirijello A, Barrio P, Gual A (2016). Treatment of alcohol use disorders in patients with alcoholic liver disease. J Hepatol.

[59] Klein CP, Kalk JF, Muting D, Klein CG (1993). The effect of alcohol on portal vein hemodynamics in nutritional-toxic liver cirrhosis. Deutsche medizinische Wochenschrift (1946).

[60] Villanueva C, Lopez-Balaguer JM, Aracil C (2004). Maintenance of hemodynamic response to treatment for portal hypertension and influence on complications of cirrhosis. J Hepatol.

[61] Muntaner L, Altamirano JT, Augustin S, González A, Esteban R, Guardia J, Genescà J (2010). High doses of beta-blockers and alcohol abstinence improve long-term rebleeding and mortality in cirrhotic patients after an acute variceal bleeding. Liver Int.

[62] Jaurigue MM, Cappell MS (2014). Therapy for alcoholic liver disease. World J Gastroenterol : WJG.

[63] Spahr L, Goossens N, Furrer F, Dupuis M, Vijgen S, Elkrief L, Giostra E, Rubbia-Brandt L, Frossard JL (2018). A return to harmful alcohol consumption impacts on portal hemodynamic changes following alcoholic hepatitis. Eur J Gastroenterol Hepatol.

[64] Argo CK, Caldwell SH (2009). Epidemiology and natural history of non-alcoholic steatohepatitis. Clin Liver Dis.

[65] Liu B, Balkwill A, Reeves G, Beral V (2010). Body mass index and risk of liver cirrhosis in middle aged UK women: prospective study. BMJ (Clinical research ed).

[66] Berzigotti A, Abraldes JG (2013). Impact of obesity and insulin-resistance on cirrhosis and portal hypertension. Gastroenterologia y hepatologia.

[67] Spengler EK, O’Leary JG, Te HS (2017). Liver transplantation in the obese cirrhotic patient. Transplantation.

[68] Bhardwaj A, Kedarisetty CK, Vashishtha C (2017). Carvedilol delays the progression of small oesophageal varices in patients with cirrhosis: a randomised placebo-controlled trial. Gut.

[69] Reiberger T, Ulbrich G, Ferlitsch A, Payer BA, Schwabl P, Pinter M, Heinisch BB, Trauner M, Kramer L, Peck-Radosavljevic M, Vienna Hepatic Hemodynamic Lab (2013). Carvedilol for primary prophylaxis of variceal bleeding in cirrhotic patients with haemodynamic non-response to propranolol. Gut.

[70] The PROVA Study Group. Prophylaxis of first hemorrhage from esophageal varices by sclerotherapy, propranolol or both in cirrhotic patients: a randomized multicenter trial. Hepatology. 1991;14(6):1016–24. 1959848

[71] Merkel C, Marin R, Sacerdoti D, Donada C, Cavallarin G, Torboli P, Amodio P, Sebastianelli G, Bolognesi M, Felder M, Mazzaro C, Gatta A (2000). Long-term results of a clinical trial of nadolol with or without isosorbide mononitrate for primary prophylaxis of variceal bleeding in cirrhosis. Hepatology.

[72] Merkel C, Bolognesi M, Sacerdoti D, Bombonato G, Bellini B, Bighin R, Gatta A (2000). The hemodynamic response to medical treatment of portal hypertension as a predictor of clinical effectiveness in the primary prophylaxis of variceal bleeding in cirrhosis. Hepatology.

[73] Abraczinskas DR, Ookubo R, Grace ND, Groszmann RJ, Bosch J, Garcia-Tsao G, Richardson CR, Matloff DS, Rodés J, Conn HO (2001). Propranolol for the prevention of first esophageal variceal hemorrhage: a lifetime commitment?. Hepatology.

[74] Turnes J, Garcia-Pagan JC, Abraldes JG, Hernandez-Guerra M, Dell’Era A, Bosch J (2006). Pharmacological reduction of portal pressure and long-term risk of first variceal bleeding in patients with cirrhosis. Am J Gastroenterol.

[75] Reiberger T, Ulbrich G, Ferlitsch A, Payer BA, Schwabl P, Pinter M, Heinisch BB, Trauner M, Kramer L, Peck-Radosavljevic M, Vienna Hepatic Hemodynamic Lab (2013). Carvedilol for primary prophylaxis of variceal bleeding in cirrhotic patients with haemodynamic non-response to propranolol. Gut.

[76] Je D, Paik YH, Gwak GY, Choi MS, Lee JH, Koh KC, Paik SW, Yoo BC (2014). The comparison of esophageal variceal ligation plus propranolol versus propranolol alone for the primary prophylaxis of esophageal variceal bleeding. Clin Mol Hepatol..

[77] Bhardwaj A, Kedarisetty CK, Vashishtha C, et al. Carvedilol delays the progression of small oesophageal varices in patients with cirrhosis: a randomised placebo-controlled trial. Gut. 2016. 10.1136/gutjnl-2016-311735 27298379

[78] Kim JH, Sinn DH, Kim K, Kang W, Gwak GY, Paik YH, Choi MS, Lee JH, Koh KC, Paik SW (2016). Primary prophylaxis for variceal bleeding and the improved survival of patients with newly diagnosed hepatocellular carcinoma. Dig Dis Sci.

[79] Reiberger T, Mandorfer M (2017). Beta adrenergic blockade and decompensated cirrhosis. J Hepatol.

[80] Garcia-Tsao Guadalupe, Sanyal Arun J., Grace Norman D., Carey William (2007). Prevention and management of gastroesophageal varices and variceal hemorrhage in cirrhosis. Hepatology.

[81] Bosch J (2013). Carvedilol: the beta-blocker of choice for portal hypertension?. Gut.

[82] Sinagra E, Perricone G, D’Amico M, Tine F, D’Amico G (2014). Systematic review with meta-analysis: the haemodynamic effects of carvedilol compared with propranolol for portal hypertension in cirrhosis. Aliment Pharmacol Ther.

[83] Mandorfer M, Peck-Radosavljevic M, Reiberger T. Prevention of progression from small to large varices: are we there yet? An updated meta-anaylsis. 2016. 10.1136/gutjnl-2016-312814 27694143

[84] D’Amico G, Pagliaro L, Bosch J (1999). Pharmacological treatment of portal hypertension: an evidence-based approach. Semin Liver Dis.

[85] Villanueva C, Aracil C, Colomo A, Hernández–Gea V, López–Balaguer JM, Alvarez–Urturi C, Torras X, Balanzó J, Guarner C (2009). Acute hemodynamic response to beta-blockers and prediction of long-term outcome in primary prophylaxis of variceal bleeding. Gastroenterology.

[86] La Mura V, Abraldes JG, Raffa S (2009). Prognostic value of acute hemodynamic response to i.v. propranolol in patients with cirrhosis and portal hypertension. J Hepatol.

[87] Hernandez-Gea V, Aracil C, Colomo A (2012). Development of ascites in compensated cirrhosis with severe portal hypertension treated with beta-blockers. Am J Gastroenterol.

[88] Groszmann RJ, Bosch J, Grace ND, Conn HO, Garcia-Tsao G, Navasa M, Alberts J, Rodes J, Fischer R, Bermann M, Rofe S, Patrick M, Lerner E (1990). Hemodynamic events in a prospective randomized trial of propranolol versus placebo in the prevention of a first variceal hemorrhage. Gastroenterology.

[89] Villanueva C, Albillos A, Genescà J (2016). Preventing the decompensation of cirrhosis with β-blockers in patients with clinically significant portal hypertension. A multicenter double-blind placebo-controlled randomized clinical trial. Hepatology.

[90] D’Amico G, Pasta L, Morabito A, D’Amico M, Caltagirone M, Malizia G, Tinè F, Giannuoli G, Traina M, Vizzini G, Politi F, Luca A, Virdone R, Licata A, Pagliaro L (2014). Competing risks and prognostic stages of cirrhosis: a 25-year inception cohort study of 494 patients. Aliment Pharmacol Ther.

[91] Serste T, Melot C, Francoz C (2010). Deleterious effects of beta-blockers on survival in patients with cirrhosis and refractory ascites. Hepatology.

[92] Mandorfer M, Bota S, Schwabl P (2014). Nonselective beta blockers increase risk for hepatorenal syndrome and death in patients with cirrhosis and spontaneous bacterial peritonitis. Gastroenterology.

[93] Serste T, Njimi H, Degre D (2015). The use of beta-blockers is associated with the occurrence of acute kidney injury in severe alcoholic hepatitis. Liver Int.

